# A randomised controlled trial on hypnotherapy for irritable bowel syndrome: design and methodological challenges (the IMAGINE study)

**DOI:** 10.1186/1471-230X-11-137

**Published:** 2011-12-20

**Authors:** Carla E Flik, Yanda R van Rood, Wijnand Laan, André JPM Smout, Bas LAM Weusten, Peter J Whorwell, Niek J de Wit

**Affiliations:** 1 Julius Centre for Health Sciences and Primary Care, University Medical Centre, Universiteitsweg 100, 3584 CG Utrecht, The Netherlands; 2 Psychiatric policlinic, Leids University Medical Centre, Post box: 9600, 2300 RC Leiden, The Netherlands; 3 Julius Centre for Health Sciences and Primary Care, University Medical Centre, Universiteitsweg 100, 3584 CG Utrecht, The Netherlands; 4 Department of Gastroenterology and Hepatology, Academic Medical Centre, Meibergdreef 9, 1105 AZ Amsterdam, The Netherlands; 5 St. Antonius Hospital, Koekoekslaan 1, 3435 CM Nieuwegein, The Netherlands; 6 Education and Research Centre, Wythenshawe Hospital, Manchester, M23 9LT, UK; 7 Julius Centre for Health Sciences and Primary Care, University Medical Centre, Universiteitsweg 100, 3584 CG Utrecht, The Netherlands

## Abstract

**Background:**

Irritable Bowel Syndrome (IBS) is a common gastro-intestinal disorder in primary and secondary care, characterised by abdominal pain, discomfort, altered bowel habits and/or symptoms of bloating and distension. In general the efficacy of drug therapies is poor. Hypnotherapy as well as Cognitive Behaviour Therapy and short Psychodynamic Therapy appear to be useful options for patients with refractory IBS in secondary care and are cost-effective, but the evidence is still limited. The IMAGINE-study is therefore designed to assess the overall benefit of hypnotherapy in IBS as well as comparing the efficacy of individual versus group hypnotherapy in treating this condition.

**Methods/Design:**

The design is a randomised placebo-controlled trial. The study group consists of 354 primary care and secondary care patients (aged 18-65) with IBS (Rome-III criteria). Patients will be randomly allocated to either 6 sessions of individual hypnotherapy, 6 sessions of group hypnotherapy or 6 sessions of educational supportive therapy in a group (placebo), with a follow up of 9 months post treatment for all patients. Ten hospitals and four primary care psychological practices in different parts of The Netherlands will collaborate in this study. The primary efficacy parameter is the responder rate for adequate relief of IBS symptoms. Secondary efficacy parameters are changes in the IBS symptom severity, quality of life, cognitions, psychological complaints, self-efficacy as well as direct and indirect costs of the condition. Hypnotherapy is expected to be more effective than the control therapy, and group hypnotherapy is expected not to be inferior to individual hypnotherapy.

**Discussion:**

If hypnotherapy is effective and if there is no difference in efficacy between individual and group hypnotherapy, this group form of treatment could be offered to more IBS patients, at lower costs.

**Trial registration number:**

ISRCTN: ISRCTN22888906

## Background

Irritable bowel syndrome (IBS) is a chronic functional gastrointestinal disorder, characterised by recurrent episodes of abdominal pain, discomfort, altered bowel habits and/or symptoms of bloating and distension, not explained by structural or biochemical abnormalities [1]. The estimated prevalence is 14-24% for women and 5-19% for men [2]. The consultation rate is relatively low: only 20-25% of IBS patients seek medical advice [3] but because the prevalence is so high this still represents a substantial number of patients. The reported incidence of IBS in primary care is 4-13/1000 patients a year [4]. General Practitioners see on average 1-2 new IBS patients a week. In the Netherlands about 10% of the patients seen by a GP are referred to a medical specialist, i.e. gastroenterologists [5], in UK 44% [1]. The estimated number of patients, who are diagnosed with IBS after referral to the gastroenterologists, varies from 20-70% [6]. Patients with IBS can have severe and often incapacitating complaints, resulting in as much annual absence from work as, for instance, from the flu [7]. The diagnosis can only be considered if there are no other indications of organic pathology and there are now consensus based criteria, the most recent of which are the so called Rome III criteria [8]:

### Irritable Bowel Syndrome

#### Diagnostic criterion*

Recurrent abdominal pain or discomfort** at least 3 days/month in the last 3 months associated with *two or more *of the following:

Improvement with defecation

Onset associated with a change in frequency of stool

Onset associated with a change in form (appearance) of stool

* Criterion fulfilled for the last 3 months with symptom onset at least 6 months prior to diagnosis

** "Discomfort" means an uncomfortable sensation not described as pain.

In pathophysiology research and clinical trials, a pain/discomfort frequency of at least 2 days a week during screening evaluation is recommended for subject eligibility

Three types of IBS can be distinguished: IBS with either predominant constipation, predominant diarrhoea, or alternating periods of diarrhoea and constipation. Several pathophysiological mechanisms underlying IBS have been proposed, including a disturbance in intestinal motility and enhanced visceral sensitivity which, according to the bio-psycho-social model of IBS, interact with other factors such as environmental influences, parent-child interactions and disturbed stress responses to result in symptoms [9].

Effective therapy of IBS is lacking. A recent review on pharmacological treatment for IBS [10] concluded that in general the efficacy of drug therapies is poor. Bulking agents, antispasmodics, and antidepressants can be tried but the response is often suboptimal.

Since 1984 [11] there has been an increasing research interest on the effectiveness of psychological treatment for IBS. A wide range of psychotherapeutic interventions have been studied including: relaxation therapy, biofeedback, cognitive behavioural therapy (CBT), short psychodynamic therapy and hypnotherapy. Two Cochrane reviews on the efficacy of hypnotherapy [2] and other psychological therapies [12] support effectiveness. In England the NICE guideline (2008) on IBS was published, with a special section on the psychological interventions which concluded that "CBT as well as short Psychodynamic and Hypnotherapy can be a useful option for patients with refractory IBS" [13]. Hypnosis is officially recognised as a legitimate medical treatment by the British Medical Association (1955) and the American Medical Association (1958). There has been much research on the use of hypnosis in the treatment of (chronic) pain [14] and, as pain is the main symptom of IBS, it is understandable that therapists have applied hypnosis in treatment of IBS patients. Pooled results of research about the effectiveness of hypnotherapy for IBS-patients are described in three reviews/meta-analysis. The NICE guideline [13] concludes that hypnotherapy may be considered a promising intervention for IBS, but judges the evidence as still too limited. Further investigation is recommended, with special interest in the potential of this intervention as a primary care therapy option, with long term follow-up. The Cochrane review on hypnotherapy for treatment of IBS concludes that "The quality of the included trials was inadequate to allow any conclusion about the efficacy of hypnotherapy for irritable bowel syndrome." And, "More research with high quality trials is needed" [2].

In a more recent meta-analysis Ford et al. conclude that hypnotherapy leads to less persistence of complaints than usual care or control therapy [15].

On the basis of these publications one can conclude that hypnotherapy is a promising and possibly cost-effective intervention for IBS in secondary care. Further investigation with high quality trials and long term follow up is needed, especially with regard to its efficacy in a primary care setting.

To improve cost effectiveness, group application of hypnotherapy could be considered. So far, there has only been one study on a group application [16], indicating no significant difference in effectiveness in a population of only 33 patients.

We have designed a RCT, comparing the effectiveness and costs of individual and group hypnotherapy with a control intervention in patients with IBS. In this paper, we describe the aim, design and methodological challenges of the study protocol.

## Methods/Design

### Aims

The primary objectives of this study are two-fold. The first aim is to assess the efficacy of hypnotherapy in IBS treatment. The second goal is to compare the efficacy of group hypnotherapy with individual hypnotherapy in IBS treatment. Secondary objectives are to assess the effect of individual or group hypnotherapy on symptom severity, on quality of life, dysfunctional cognitions, psychological complaints, self-efficacy, and IBS related costs.

### Design

**Figure 1:**
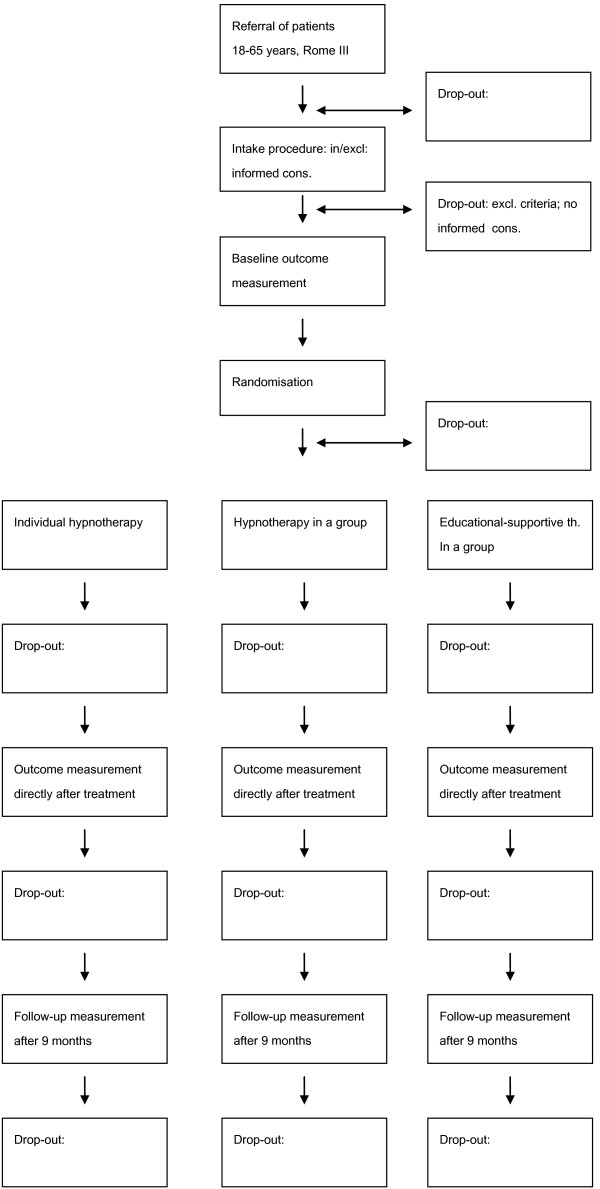
**Design of the RCT**.

The study is designed as a comparative and a non-inferiority 12-weeks single blind controlled parallel-group trial. The trial will involve IBS patients in primary and secondary care, who will be randomly allocated to either 6 sessions of individual hypnotherapy, 6 sessions of hypnotherapy in group format, or 6 sessions of educational supportive therapy in a group format (control condition) (Flowchart Figure 1). Starting May 2011, the inclusion of patients will take approximately two years, with a follow-up of nine months.

### Ethical considerations

The study is conducted according to the principles of the Declaration of Helsinki and in accordance with the Medical Research Involving Human Subjects Act (WMO). The study protocol has been approved by the Medical Ethics Committee of the University Medical Centre of Utrecht, the Netherlands.

Patient data will be coded and analysing and publication of the results will be anonymous.

### Hypotheses

The primary hypotheses assessed in this study are:

At the end of therapy, more patients in the hypnotherapy condition will report adequate relief than in the educational supportive therapy condition (control treatment).

Hypnotherapy offered in a group format is as effective as individual hypnotherapy.

### Study population

The study population consists of patients with IBS referred by GP or medical specialist. Ten general hospitals and four psychological practices in primary care collaborate in the study.

#### Inclusion criteria

Age 18-65 years

diagnosis of IBS according to the Rome III criteria. The Dutch translated version of the IBS Module of the Rome Foundation [8] is used to check criteria.

#### Exclusion criteria

inability to understand the content of the sessions, because of insufficient command of the Dutch language.

inability to fill out the questionnaires.

inability (for example: too aggressive) or unwillingness to function in a group.

a psychiatric condition that requires attention first (for example severe depression, PTSS or psychosis).

a combination of IBS and other chronic bowel diseases, as far as they already diagnosed, such as ulcerative colitis, Crohn's disease or coeliac disease.

major surgery to the lower gastrointestinal tract, such as partial or total colectomy, small bowel resection or partial or total gastrectomy.

past or present radiotherapy to the abdomen (eg carcinoma of the cervix).

### Procedure

Cooperating GPs and specialists are asked to give each eligible patient an invitational letter explaining the goal of the study and describing the interventions. The patient fills in the IBS questionnaire to confirm the diagnosis. If the patient is interested in participating in the trial, the GP or specialist hands the patient study information with the Informed Consent letter and a brochure from the government explaining the law and the rights of patients participating in medical research. After confirmation of the intention to participate, the patient is referred to the collaborating psychologist, either in the hospital or in primary care practice. The psychologist checks the in- and exclusion criteria and explains the study. If the patient is willing to participate, she/he signs the Informed Consent letter, completes the questionnaires and sends them, together with the IBS-criteria checklist in a reply-envelope to the investigator. After randomisation the patient is invited to the therapy she/he is randomised to.

### Randomisation

After inclusion, patients are randomly allocated to one of the three treatment conditions by means of a computer-based, six-block random number tables procedure. Because for group treatment six patients are required, the randomisation is done block-wise to prevent prolonged waiting time for the individual patient. The researcher performs randomisation.

### Intervention

Patients will be randomised to one of the three treatment arms.

(a) Individual hypnotherapy is delivered in a series of 6 individual, bi-weekly 45-minute sessions in which patients receive a structured hypnotherapy treatment. The treatment procedure is developed by the investigator (C.F.) based on the hypnotherapy protocol for IBS from the research group of Whorwell in Manchester UK [17]. Basically, hypnotic suggestions are given to normalise motility of the gut and reduce pain and feelings of discomfort. The precise wording is adjusted to the individual patient. Treatment is given by qualified psychologists who are educated as hypnotherapists and specifically trained for the intervention.

(b) Group hypnotherapy is delivered in a series of 6 bi-weekly 60-minute group sessions, with a maximum of 6 IBS patients per group. The group hypnotherapy is based on the same principle as the individual hypnotherapy but is adapted for the group format. Group hypnotherapy will be given by the same psychologists who deliver the individual therapy.

Both individual and group hypnotherapy patients are given homework assignments consisting of CD recorded hypnotherapeutic exercises. Carrying out these exercises takes 15-20 minutes, at least once daily.

(c) Educational supportive therapy is delivered in a series of 6 bi-weekly 60-minute group sessions, with a maximum of 6 patients per group. In the sessions topics are discussed that are of importance to IBS patients, as determined by research [18-22]. The topics include: information about IBS; the role of food and life-regulation and dealing with stress in managing IBS. Homework assignments are given that take about 15-20 minutes per day.

Educational supportive therapy will be performed by nurse practitioners or psychological assistants who are specifically trained for the intervention.

Treatments are carried out according to a detailed therapy protocol in which all sessions are described (verbatim). Therapists receive this verbatim protocol, the CD with the hypnotherapeutic exercises and homework assignments.

All therapists are trained in the protocol they carry out and subsequent treatment is supervised by the principal researcher.

To prevent contamination of groups, therapists giving hypnotherapy will not give educational supportive therapy and vice versa.

### Other treatments during the study

Patients may continue usual care as instructed by their physicians but are asked not to change it during the research, except on doctor's advice. They are free to seek other treatment. This will be recorded in their questionnaire.

### Study parameters/endpoints

As yet, it is not known what makes hypnotherapy an effective treatment. We assume that hypnotherapy has a direct influence on visceral hypersensitivity (pain processing and pain perception [14] and an indirect influence on pain perception through relaxation and changing cognitions. Furthermore, gut motor activity (motility) can be influenced by hypnotherapy [14]. Finally, hypnotherapy can have an effect on psychological factors such as self-efficacy and feelings of depression that can play a role in IBS. Our choice for outcome measures has been influenced by these assumptions.

#### Main study parameter/endpoint

In line with previous conclusions on optimal outcome assessment in trials on functional gastrointestinal disease [23,24], we chose the number of weeks with adequate symptom relief as the primary outcome. This measure addresses weekly symptom improvement in IBS with treatment using a single question ("Did you have adequate relief of IBS-related abdominal pain or discomfort in the past week?") scored on a dichotomous scale (Yes/No). This instrument is a well validated simple outcome assessment for IBS treatment [25] with a positive responder being defined as someone with more than 2 weeks adequate relief per month [26] which has determined to indicate a clinically significant improvement [26].

The first primary outcome is the difference in the percentage of positive responders between group hypnotherapy and group educational therapy. Group hypnotherapy is expected to be substantially more effective than educational therapy.

The second primary outcome is the difference in percentage of positive responders between group hypnotherapy and individual hypnotherapy. Group hypnotherapy is expected not to be inferior to individual hypnotherapy.

#### Secondary study parameters/endpoints

##### Irritable Bowel Syndrome Symptom Severity

IBS symptoms will be monitored using the IBS symptom severity score (IBS-SSS).

The IBS-SSS assesses five features of IBS (pain severity and frequency; abdominal distension; bowel satisfaction; interference with life in general) and their intensity, using visual analogue scales [27]. The IBS-SSS has been validated and its use is recommended in an overview on outcome measures [24].

##### Irritable Bowel Syndrome Quality of Life

Disease-specific quality of life will be assessed using the Irritable Bowel Syndrome Quality of Life scale (IBS QOL) [28]. It has been validated in different populations. This instrument includes 30 items and consists of nine scales (dysphoria; interference with activity; body image; health worry; food avoidance; social reaction; sexuality; relationships; overall scale).

#### Other measurements

##### Psychological symptoms

Psychological symptoms are assessed with the Dutch version of the Symptom Checklist (SCL-90). It was originally published by Derogatis [29] and translated and validated for the Dutch population by Arrindell & Ettema [30] The SCL-90 is a 90-item multidimensional self-report inventory, designed to evaluate a broad range of psychological problems and symptoms of psychopathology. It has 9 subscales: Agoraphobia, Anxiety, Depression, Somatisation, Insufficiency of thought and action, Distrust and Interpersonal Sensitivity, Hostility, Sleeping problems and Psychoneuroticism (total score).

The SCL-90 is an internationally accepted, widely used, questionnaire with good psychometric qualities[29].

##### Dysfunctional cognitions

The Cognitive Scale for Functional Bowel Disorders (CS-FBD) has been developed by Toner [31] and translated with permission from the author by van Rood. The CS-FBD consists of 31 items to measure a patient's level of dysfunctional cognitions concerning his or her IBS. It is a valid and reliable scale that can be used as an outcome measure in evaluating the efficacy of psychotherapeutic interventions for functional bowel disorders [31].

##### Self-efficacy

The Self-Efficacy Scale (SES) is a seven-item questionnaire to measure the confidence of patients about their capacity to influence their somatic complaints. It was originally developed for patients suffering from chronic fatigue [32] and adapted with permission of the author by C.Flik and Y. van Rood for patients with IBS.

##### Costs

The Trimbos/iMTA Questionnaire (Tic-P) for costs associated with Psychiatric Illness, measures direct medical costs due to health care utilization during the past four weeks and indirect non-medical costs due to productivity loss during the past two weeks[33]. It can be adapted for other conditions as well and for this study was adapted for IBS.

### Time line and follow-up

Enrolment started May 2011. The inclusion of patients will take approximately two years. After the active treatment period of 12 weeks, all patients will be followed for an additional period of 9 months, to assess the sustainability of the effects of the interventions. The primary outcome will be measured weekly during the first four weeks after finishing treatment and again weekly during four weeks 9 months post treatment. The secondary endpoints will be assessed prior to intervention, immediately after the intervention and at 9 months after finishing the intervention.

### Other study parameters

Patients are given a "CD use" diary in which they can record the number of times they used the CD or did the hypnotherapeutic exercises in the last week. The homework assignments in the educational supportive therapy are not registered separately.

**Table 1:** Overview of assessments.

	Weeks					
**Visit** **(time point)**	**0** **(start of treatment)**	**12** **(end of treatment)**	**12+** **Every 4 week** **(end of treatment + 4 weeks)**	**12+** **Every week** **After treatment**	**52** **(9 months after treatment)**	**52 + 4** **(4 weeks after 9 months)**

Assessments						

IBS-Mode	X				X	

Informed consent	X					

Inclusion exclusion criteria	X					

Demographic data	X					

Status report	X					

Adequate relief			X			X

IBS-SSS	X	X			X	

IBS-QOL	X	X			X	

SCL-90	X	X			X	

CS-FBD	X	X			X	

SES	X	X			X	

TiC-P	X	X			X	

CD-diary		X		X		

### Sample Size Calculation

Assuming an individual hypnotherapy response rate of 57% [2], a maximum acceptable level of difference of 15% between individual and group hypnotherapy, an alpha of 0.05 and power (1-beta) of 0.80, 135 patients are required in both arms of the non-inferiority trial to show that group-hypnotherapy is not inferior to individual hypnotherapy.

Spiller [34], in an overview of 25 randomised controlled trials, states that when the placebo response is plotted against the length of study, placebo response will be maximum of 75% at around 6-8 weeks, falling to 25% at 24 weeks and zero at 12 months. Our follow-up period will be 9 months. To test whether group hypnotherapy is more effective than group placebo therapy, we will have more than sufficient power assuming a placebo-response rate of 25% and a group hypnotherapy response rate of 57% [13] using the proposed 135 patients in both arms. Powering the study for only the comparison between hypnotherapy and the control intervention, would require only 44 patients in both arms assuming an alpha of 0.05, a power (1-beta) of 0.80, a cluster size of 6 patients per therapist and an intra-class correlation coefficient of 0.05. Assuming at least 10% of loss to follow-up, 354 patients need to be included in the study (150+150+54 (placebo-arm)).

### Recruitment

The 354 patients are recruited in primary and secondary care.

Primary care: Assuming an estimated mean incidence of IBS in primary care in 8 of every 1000 patients, 20 newly diagnosed patients per practice per year and an expected inclusion rate of 25%, 30 general practitioners, each referring 5 patients each year, will be able to refer the total required number of patients (354) in 2,5 years.

Secondary care: The estimation of the number of consultations for IBS in gastroenterology practice, varies between 20-70% of all referrals per year. Using a conservative estimate, about 25% of all referrals will be because of IBS. Thus, in a normal gastroenterologist's practice of a general hospital, with 500 new patients for each gastroenterologist each year, there will be 125 new IBS patients each year. Assuming that 10% of the patients will be interested in taking part in the trial, each gastroenterologist can refer 12 patients a year. Therefore 10 hospitals with at least 2 referring specialists to cooperate in this study for 1,5 years (354 patients) are needed.

Ten hospitals and four primary care psychological practices (working with several referring GPs), in different parts of the Netherlands, have agreed to collaborate in the trial. This ensures that the study population will be representative of the whole Dutch population.

When combining the expected referral rates from the primary and secondary care practices, two years of recruitment will be sufficient to attain the required number (354) of participants.

### Statistical analysis

#### General remarks

All statistical analyses will be based on the intention-to-treat principle, i.e. patients will be analysed according to their initial assignment to one of the randomisation arms.

#### Uni- & bivariate analysis

The percentage of patients with a positive response will be calculated post-treatment and at 9 months after treatment and differences between the three randomisation arms will be tested with the chi-square test.

In addition, the adequate relief scores and secondary outcomes (IBS- SSS, IBS-QoL, SCL-90-, CS- FBD-, SES- and TiC-P scores) will be estimated by time point of assessment and arm of randomisation using a statistical model for repeated measurements (preferably a random effects model). To assess the difference between the arms of randomisation by time point of assessment, terms for interaction of {arm × time point} will be included in the statistical model.

#### Multivariate analysis

##### Subgroup analyses

The analyses described above will also be performed separately for patients who fulfil the Rome III IBS diagnostic criteria for constipation-predominant, diarrhoea-predominant and mixed type IBS. In a study of Gonsakorale et al [35] one of the conclusions was that hypnotherapy was less useful for males with diarrhoea. If males with diarrhoea-predominant bowel habit are over-represented in one of the conditions, adjustment in multivariate regression analysis is indicated.

Analyses will be performed separately for those referred from primary and secondary health care. In secondary care, IBS-patients have a longer disease history, more psychosocial co-morbidity and higher stress and depression scores. It is possible that these differences may influence the effect of the intervention.

#### Adjustment for confounders

In case of (unexpected) differences in relevant baseline characteristics between the three comparison groups, adjustment for the possible confounding effect of these differences will be performed in multivariate regression analyses.

### Risk/Benefit for patients

Benefit: Research indicates that hypnotherapy is a promising intervention for IBS which could potentially be of considerable benefit to the patients participating in the study. Research also indicates that patients want to be informed more about IBS. Patients randomised to the placebo condition receive this information and thus can also benefit from participating in the study.

Risk: In all the research that has been evaluated by the Cochrane study, no adverse effects for patients have been reported. This treatment is symptom-oriented with care taken to exclude those individuals with contra-indications and treatment is always carried out by experienced therapists educated in hypnotherapy.

## Discussion; Methodological challenges

In planning this study, a number of challenges commonly encountered in studies on functional GI diseases needed to be overcome: selection of the study population; diagnostic inclusion criteria; a valid control intervention; placebo effect and outcome assessment.

### Selection of the study population

Lack of heterogeneity among IBS patients can affect the generalisability of trial results [36]. To overcome this problem, patients will be recruited from primary and secondary care populations. The NICE guideline (2008) recommends the inclusion of patients from primary care to enhance the generalisability of the results. Co-operating hospitals and primary care practices are situated all over Holland, so the population participating in the study will be representative of the whole Dutch population.

Although attention to the study was drawn by announcements to both physicians in primary and secondary care and patients by the Dutch IBS Patient Foundation web-site, there will always be a selection bias, because only patients who are motivated are included in this study. It cannot be ruled out that they differ in some respects from those who do not want to participate on the effect of hypnotherapy.

### Diagnostic inclusion criteria

The selection of the diagnostic inclusion criteria were a second challenge in the study. A task force of the American Journal of Gastroenterology [37] conducted a systematic review on the accuracy of symptom-based criteria in the diagnosis of IBS. They summarise the diagnostic criteria that have been used over the years which are those reported by: Manning (1978); Kruis (1984) Rome I (1990); Rome II (1999) Rome III (2006). The main differences are in the number of symptoms included as well as their duration which is important as they should be been present for a considerable period of time before the diagnosis is considered.

The accuracy of the Rome II and III criteria has not been formally evaluated yet and the ACG task force chose their own pragmatic definition: "abdominal pain or discomfort that occurs in association with altered bowel habits over a period of at least three months".

Because of the problem of the absence of a specific diagnostic test for IBS, the Rome committee developed the IBS-module, a short questionnaire (10 questions) with a scoring device. It is applicable in primary and secondary care and takes only a few minutes for patients to complete. For standardisation and to facilitate comparison of research populations in the future, in this RCT the questionnaire was chosen to confirm the diagnosis of IBS.

The Rome committee has started a translation project to make it possible for this questionnaire to be used worldwide. Following official translation guidelines [38] and with the official consent of the Rome III committee, the module has been translated into Dutch and will be of use to every specialist in the field of IBS in the Netherlands.

### Optimal control intervention

To design a good control intervention is notoriously difficult in research on the effectiveness of psychological treatments [39]. "Care as usual "is not a good control-option since it does not exclude the possibility that treatment effect is due to differences in therapist attention rather than to the intervention [23]. A good placebo condition needs to have all components of the experimental intervention, except the active component. This is very difficult to realise with psychotherapeutic interventions. A "sham" intervention with the same time investment for patient and therapist but with a non therapeutic intervention, potentially generates a negative effect. Consequently, an intervention was designed in which hypnotherapy was missing but all other components were retained: time, attention, active intervention and contact with therapist. The intervention, an informative educational programme, covers topics IBS patients like to have more knowledge about [18-22]. The informative educational programme will be given by nurse practitioners or welfare workers and not by hypnotherapists because it is anticipated that hypnotherapists will automatically use the suggestive language they use in the hypnotherapy. To emphasise the supportive and educational character of this control-intervention, the deliberate use of nurse practitioners or welfare workers was felt to be the most appropriate to deliver this intervention. Furthermore, the effects of the doctor-patient relationship will be controlled for by using multiple, experienced therapists [39].

### Placebo effect

The placebo response causes serious problems for the design of RCT's in IBS. Spiller (1999) describes on the basis of 25 RCTs on medication and fibre from 1976-1998, a median placebo response of 47% [34]. Ford & Moayyedi (2010) estimated a response rate of 37.5% across all RCT's on pharmacological therapies in adult IBS-patients [40]. Spiller claims that the placebo response diminishes after approximately 12 weeks and was lost altogether by 6 months. Recommendations to diminish the effect of placebo response on outcomes of RCT's are:

Lengths of the therapy should be more than 8 weeks and follow-up for more than 6 months; diagnosis should be based on the Rome-criteria and not on clinical judgement; patient-reported endpoints are better than physician-reported outcomes; it is important to be able to distinguish between subgroups of IBS-C, IBS-D and IBS-M.

In the design of this RCT these recommendations were met in terms of: the length of therapy is 3 months; follow up will be 9 months after ending the therapy and we chose for the Rome III IBS-module to diagnose the cases and distinguish the sub-groups.

### Outcome assessment

As no objective standards for diagnosing functional GI-diseases exist, outcome assessments have to be based on subjective criteria. According to Irvine [23] the "adequate relief" questionnaire [25] is the current standard primary outcome measurement in treatment trials of Functional Gastrointestinal Disorders. For a more detailed IBS symptom assessment the IBS-Severity Scoring System [27] is recommended [24]. Furthermore, IBS related Quality of Life is an important secondary outcome measure [23]. At present, the IBS-Quality of Life measurement [28] is the best choice, "because it has been the most extensively validated and shows appropriate psychometric quality" [24]. Mangel, (outcome measure:"adequate relief "[25]) and Whorwell (IBS-SSS [27]) gave their consent for translating these questionnaires into Dutch.

IBS-complaints can have an episodic course and in accord with the recommendation on measuring severity during episodic symptoms by the research design of the Rome committee [23], patients will fill in the assessments on two occasions: immediately at the end of treatment and after nine months. Also the 'adequate relief' question is asked every week, for a period of four weeks.

It is possible that the patients in the control group with educational supportive therapy also will experience some improvement in their complaints, but the expectation is that treatment with hypnotherapy will be superior.

### Conclusion

The results of this primary and secondary care based randomised placebo controlled trial, evaluating the efficacy of individual and group hypnotherapy treatment in IBS, will contribute to the scientific basis of IBS management. The trial intends to include the greatest population of patients of all the trials in psychological treatments for IBS to date [2,12].

If the results of the study show that (group) hypnotherapy for IBS is effective, implementation into clinical practice will be the next aim.

## Competing interests

The authors declare that they have no competing interests.

## Authors' contributions

CF and NdW developed the original idea for the study. All authors contributed to the developing of the study protocol. WL substantially contributed to randomization and the statistical methods. CF developed the verbatim hypnotherapy-protocol on basis of the protocol of PW and the Educational supportive therapy in close collaboration with YvR. All authors participated in the design of the study and development of research protocols. All authors contributed to and approved the final manuscript.

## Pre-publication history

The pre-publication history for this paper can be accessed here:

http://www.biomedcentral.com/1471-230X/11/137/prepub
